# Reducing wait times and avoiding unnecessary use of high-cost mental health services through a Rapid Access and Stabilization Program: protocol for a program evaluation study

**DOI:** 10.1186/s12913-024-10697-7

**Published:** 2024-02-27

**Authors:** Medard K. Adu, Raquel da Luz Dias, Gloria Obuobi-Donkor, Ngozi Ezeanozie, Sanjana Sridharan, Jason Morrison, Patryk Simon, Bryanne Taylor, Monica MacKinnon, Shiloh Gossen, Mahmoud Awara, Mattew White, Reham Shalaby, Belinda Agyapong, Ejemai Eboreime, JianLi Wang, Cindy Feng, Lori Wozney, Prosper Koto, Jordan Warford, Gail Tomblin Murphy, Vincent Israel Opoku Agyapong

**Affiliations:** 1 Department of Psychiatry, Faculty of Medicine, Dalhousie University, Halifax, NS Canada; 2 Mental Health and Addictions Program, Nova Scotia Health, Halifax, NS Canada; 3 Research, Innovation and Discovery, Nova Scotia Health, Halifax, NS Canada; 4 Department of Psychiatry, Faculty of Medicine and Dentistry, University of Alberta, Edmonton, AB Canada; 5 Department of Community Health and Epidemiology, Faculty of Medicine, Dalhousie University, Halifax, NS Canada; 6 Mental Health and Addictions Program, IWK Health, Halifax, NS Canada

**Keywords:** Mental health, Rapid access, Stabilization, Emergency admission, Health service utilization

## Abstract

**Background:**

Emergency psychiatric care, unplanned hospital admissions, and inpatient health care are the costliest forms of mental health care. According to Statistics Canada (2018), almost 18% (5.3 million) of Canadians reported needing mental health support. However, just above half of this figure (56.2%) have reported their needs were fully met. In light of this evidence there is a pressing need to provide accessible mental health services in flexible yet cost-effective ways. To further expand capacity and access to mental health care in the province, Nova Scotia Health has launched a novel mental health initiative for people in need of mental health care without requiring emergency department visits or hospitalization. This new service is referred to as the Rapid Access and Stabilization Program (RASP). This study evaluates the effectiveness and impact of the RASP on high-cost health services utilization (e.g. ED visits, mobile crisis visits, and inpatient treatments) and related costs. It also assesses healthcare partners' (e.g. healthcare providers, policymakers, community leaders) perceptions and patient experiences and satisfaction with the program and identifies sociodemographic characteristics, psychological conditions, recovery, well-being, and risk measures in the assisted population.

**Method:**

This is a hypothesis-driven program evaluation study that employs a mixed methods approach. A within-subject comparison (pre- and post-evaluation study) will examine health services utilization data from patients attending RASP, one year before and one year after their psychiatry assessment at the program. A controlled between-subject comparison (cohort study) will use historical data from a control population will examine whether possible changes in high-cost health services utilization are associated with the intervention (RASP). The primary analysis involves extracting secondary data from provincial information systems, electronic medical records, and regular self-reported clinical assessments. Additionally, a qualitative sub-study will examine patient experience and satisfaction, and health care partners' impressions.

**Discussion:**

We expect that RASP evaluation findings will demonstrate a minimum 10% reduction in high-cost health services utilization and corresponding 10% cost savings, and also a reduction in the wait times for patient consultations with psychiatrists to less than 30 calendar days, in both within-subject and between-subject comparisons. In addition, we anticipate that patients, healthcare providers and healthcare partners would express high levels of satisfaction with the new service.

**Conclusion:**

This study will demonstrate the results of the Mental Health and Addictions Program (MHAP) efforts to provide stepped-care, particularly community-based support, to individuals with mental illnesses. Results will provide new insights into a novel community-based approach to mental health service delivery and contribute to knowledge on how to implement mental health programs across varying contexts.

**Supplementary Information:**

The online version contains supplementary material available at 10.1186/s12913-024-10697-7.

## Background

### Mental health and the burden on health services utilization and health outcomes

Emergency psychiatric care, unplanned hospital admissions and inpatient health care are the costliest forms of mental health care [1]. The frequency of Emergency Department (ED) presentations with mental health concerns and length of psychiatric hospitalizations can have significant physical, psychological, and financial consequences for patients and their families and affect the infrastructural, human, and economic well-being of healthcare systems [2, 3]. This is especially true in the era of the COVID-19 pandemic when already limited services have been further stretched by growing hospital admissions, ED presentations and an increase in extended stays [4–6]. The World Health Organization reported in October 2020 that the pandemic had disrupted or halted critical mental health services in 93% of countries worldwide [7]. Consequently, the increased demand for mental health support has renewed the interest in seeking out solutions to mitigate avoidable hospital readmissions and ED visits, as well as to lower lengths of stay (LOS) in acute care facilities [8].

People with psychiatric disorders have the highest early readmission rates among all hospitalized patients [9–11]. Early readmission is defined as readmission within 30 days of previous discharge [9]. Although deinstitutionalization of care and transition to community-based mental health care has been an approach of focus for decades [12, 13], early hospital readmission rates remain high. In Nova Scotia, the 30-day re-admission rate for inpatient mental health treatment was 10.14% for the 2021/2022 fiscal year. The unmet need for psychological treatment and the limited human resources to address this gap is a major cause of readmission in acute psychiatry units [9]. The readmission rate is a prevalent indicator used for quality assessment of care and a focus of interest for health sector policymakers [14, 15]. In psychiatry, readmission rates are usually commensurate with relapse or complications following an inpatient stay. While it may reflect premature discharge from inpatient psychiatric units, it may also relate to the lack of coordination of post-discharge healthcare services [16, 17].

Similar concerns are related to frequent ED visits. Frequent ED visitors are commonly defined as people having 4 or more ED visits during the past 12 months [18], and they are more likely to suffer from chronic somatic diseases, drug and alcohol abuse and acute mental illness [18]. Predictors for recurrent ED visits due to acute mental health problems are usually substance abuse, single status and homelessness [19].

In addition to readmission rates and frequency of ED visits, length of stay (LOS) is another important quality of care indicator. LOS is defined as the number of days between admission and discharge dates for each admission experienced [20]. LOS is likely to be multifactorial, but some factors associated with longer LOS have been consistently identified: biological sex (being male), ethnicity (being Asian, Black, or having mixed background), accommodation status (being homeless) and having the primary diagnosis of psychosis. Although there is no ideal LOS, current international recommendations advocate for an early discharge as soon as stabilization is successful, with continuation of treatment in less restrictive environments [20]. On the contrary, concerns about prioritizing shorter stay admissions include increasing medical negligence and favouring the revolving door cycle, which can be aggravated with a history of repeated admission and frequent ED visits [20, 21].

Besides the high rates of health services utilization, mental disorders are associated with major social and economic consequences for patients and their families. Patients with mental disorders have high mortality rates [22], poor quality of life [23], lower self-esteem [24], and lack of educational and income-generating opportunities, thus limiting their chances of economic independence and depriving them of social networks and status within the community [14]. Individuals with mental disorders also experience a variety of chronic physical health problems, such as hypertension and cerebrovascular diseases [15]. Despite the pervasive need for mental health treatment among individuals with mental disorders, it is generally acknowledged that many do not use healthcare services [25].

According to Statistics Canada (2018), almost 18% (5.3 million) of Canadians reported their need for some mental health support, but just above half of this figure (56.2%; 3 million) have reported their needs were fully met, while the rest (43.8%, 2.3 million) have stated their needs were partially met or unmet altogether, particularly when considering those who do not have a regular health care provider [26]. Provincially, Nova Scotia is similar to these nationally reported figures concerning the mental health care gap [27]. The greatest unmet needs reported in the province were the lack of counselling and the service cost [26, 27].

Implementation of easily accessible early intervention programs that can help prevent the frequent use of high-cost services such as ED and inpatient treatments is needed. Early intervention programs have been shown to improve mental health outcomes for individuals and their families improve quality of life, reduce disability, and increase productivity for the affected individuals [28]. There is, therefore, a pressing need to provide accessible early intervention mental health programs in flexible yet cost-effective ways. These programs can be incorporated into stepped models of mental health care, where clients receive rapid comprehensive mental health assessment prior to being matched to services that meet their needs [29]. The stepped care model includes easily accessible early intervention programs that widen access to care by offering the least restrictive and least costly interventions to most people, improving access to mental health services through better allocation of scarce resources, reducing wait times for clients and avoiding unnecessary use of high-needs/high-cost mental health services (i.e., inpatient, ED visits) [29–31].

### Rapid access and stabilization program

Nova Scotia Health (NSH) has been working to expand access to quality addiction and mental health programs through various services and technology-based health initiatives [32]. The provincial Mental Health and Addictions Program (MHAP) is delivered using the stepped care model, where a continuum of services and service providers are available to support people’s specific needs, from the least intensive interventions (health promotion, primary care, self-management, community care) to the most intensive treatment services (formal and specialized mental health & addictions care) for more complex needs [32]. To further expand capacity and access to mental health care, the province has launched a novel mental health initiative for people in need of mental health care at various intensity levels to reduce wait times for access to mental health and psychiatric support, reduce ED visits for mental health concerns and reduce inpatient psychiatric treatments.

**Fig. 1:**
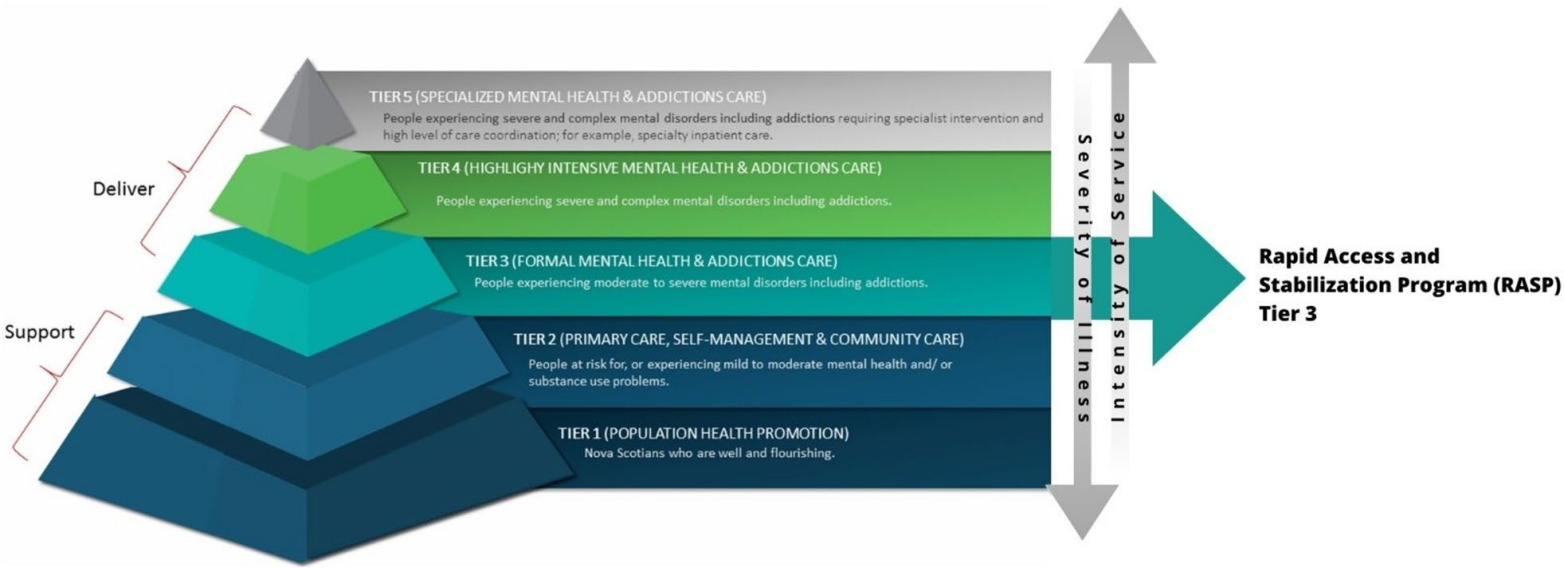
Nova Scotia Health Mental Health and Addictions Program Stepped Care Model

This new service is referred to as the Rapid Access and Stabilization Program (RASP) [33], a tier 3 model of care (Fig. 1) implemented in April 2023, which aims to offer comprehensive mental health assessment, develop treatment plans, and provide short-term stabilization and mental health support to patients.

Primary Healthcare Providers (PHP) such as mental health clinicians, nurse practitioners and general practitioners, including those from walk-in-clinics, can refer patients to the service by using the existing central intake pathway. When fully implemented, the program will also welcome walk-in patients who would receive an assessment from a mental health clinician, and if necessary, referred to one of the program psychiatrists for further assessment. Additionally, psychiatrists participating in the program will provide telephone consultations and support for PHPs across the province. All patients accessing the RASP are scheduled to arrive at the clinic 30 minutes before their appointment with the psychiatrists to complete a range of standardized assessments, which are detailed in the section for data collection below. In addition, following the psychiatric consultation, each patient is offered the opportunity to provide feedback about their experience with the RASP provider by completing either a paper-based or an online satisfaction survey. A link for the online satisfaction survey is sent via text message to the patient’s cell phone after the psychiatric evaluation. Additionally, patients can opt-in to participate in an optional Text4Support randomized controlled trial (RCT) where they can either receive daily supportive text messages which are tailored to their primary presenting problem or a single text message which has a link to the NSH/MHAP suite of e-mental health programs [34]. After each psychiatric assessment, a detailed report which covers all essential elements of a comprehensive psychiatric assessment (i.e., presenting complaints, history of presenting complaints, medication history, past psychiatric history, medical and surgical history, family history of mental illness, drug and alcohol history, forensic history, personal history, current social circumstances, pre-morbid personality and a description of the mental status following an examination) is returned to the referring primary care provider within 24 hours. The report includes scores for standardized rating scales and their interpretation and a comprehensive treatment plan based on the biological, psychological and social model. The report encourages the receiving primary care provider to contact the psychiatrist at RASP through the program phone number if they have any follow-up questions. Providers are also advised to re-refer the patient back to the program for further evaluation if the patient's mental health issues are not fully resolved after exhausting the comprehensive treatment plan and recommendations offered. The RASP psychiatrist or a clinical coordinator (mental health clinician) offers each patient information about community resources and support organizations they could utilize in their recovery journey. They are also offered psychoeducation to improve their mental health literacy and information on lifestyle changes that can promote good mental health, such as an increase in physical exercise, good nutrition, avoidance of substance and alcohol abuse and use of self-help resources. Patients with complex presentations or deemed by the RASP psychiatrists to require further psychiatric or mental health support are transferred to the community mental health program for follow-up. In addition, patients accessing the RASP who present an acute risk of harm to themselves or others are transferred to the hospital Emergency Department for further assessment and consideration for inpatient treatment.

In this paper, we present the protocol for a service evaluation study that aims to assess the effectiveness and impact of the RASP on various aspects, including high-cost health services utilization, clinical outcomes, healthcare partners' perceptions, and patient satisfaction. The study employs a research hypothesis-driven approach to comprehensively evaluate the program's performance and potential benefits for individuals accessing mental health services and for the health system`. Additionally, we aim to identify and analyze the sociodemographic and clinical characteristics of the assisted population to gain a deeper understanding of the program's reach and effectiveness. Through this research, we seek to provide valuable insights that can contribute to improving and optimizing mental health service delivery models, ultimately enhancing patient care and outcomes.

## Methods

### Study objectives

The primary objective of this study is to evaluate the effectiveness and impact of RASP on high-cost health services utilization (e.g. ED visits, mobile crisis visits and inpatient mental health utilization). To achieve this objective, we will conduct a within-subject comparison by analyzing health services utilization data from patients attending the RASP program one year pre- and post-assessment. Additionally, a between-subject comparison will be made using historical data from a control population consisting of patients referred by a PHP to see a mental health clinician before RASP implementation. Another primary objective is to assess qualitative data from healthcare partners' perceptions related to the impact of the RASP on access to community mental health care for patients and support for primary care providers, as well as to determine patient experiences and satisfaction with the program.

The secondary objectives of this study are to i) identify the sociodemographic characteristics of the population assisted by the RASP, ii) evaluate the prevalence and correlates of the various psychological conditions of the assisted population, iii) assess recovery, well-being, and risk measures in the assisted population at the program entry, and iv) compare health services utilization in the subset of patients who opt to participate in the Text4Support RCT with those who do not.

The study seeks to address the following research questions:

A. Effectiveness and Impact on Health Services Utilization of the RASP:
  1. Did RASP increase the volume of patients referred by PHP? Measured by the number of patients referred by a PHP for a psychiatrist consultation before and after RASP implementation.
  2. Did RASP increase the proportion of patients triaged by Central Intake services who had direct access to psychiatric consultations? Measured by the proportion of referrals received by central intake that were booked directly for psychiatry consultation, before and after RASP implementation.
  3. Did RASP reduce wait times for access to psychiatrists in the publicly funded MHAP? Measured by the number in calendar days waiting from date referral was received by Central Intake to date patient is scheduled for a psychiatric consultation before and after RASP implementation.
  4. Did RASP improve efficiency of the Community Mental Health Programs (CMHP) to address the mental health needs of patients with complex mental health needs? Measured by wait-times for assess to psychiatrists within the CMHP following a referral by a mental health clinician before and after RASP implementation.
  5. Did RASP reduce the volume of patients referred by PHP to the ED for a psychiatric consultation? Measured by the number of patients referred by a PHP for a psychiatrist consultation at the ED before and after RASP implementation.
  6. Did RASP reduce ED visits, mobile crisis visits, and inpatient mental health services utilization? Measured by the number of mental health and addictions (MHA)-specific ED visits, number of mobile crisis visits, number of MHA-specific hospital admissions, and number in calendar days in LOS for Central Zone patients, before and after RASP implementation.
  7. Did patients who attended the RASP and opted to participate in the Text4Support RCT differ in their health services utilization compared to patients who opted not to participate in the Text4Support RCT? Measured by the number of MHA-specific ED visits, number of mobile crises calls, number of MHA-specific hospital admissions, and LOS in calendar days, one-year post-RASP assessment.
  8. What are the costs and benefits to Nova Scotia Health from having a RASP for mental health? Measured by the net cost savings associated with RASP considering costs associated with the program and costs saved from avoided high-cost health services utilization (ED visits, mobile crisis visits, and inpatient treatments).
B. Perspectives and Experiences of Healthcare Partners and Patients
  1. What are healthcare partners' perceptions related to the impact of the RASP on access to community mental health care for patients and support for primary care providers? Measured by qualitative data from key informant interviews with program directors, psychiatrists, primary healthcare providers, MHA service leaders, and representatives from community organizations.
  2. What are the patients' perceptions related to their overall experience and satisfaction with the service received? Measured by quantitative and qualitative data from the RASP's satisfaction survey and qualitative data from focus group sessions with a sub-group of the assisted population.
C. Population Characteristics and Mental Health Measures
  1. What proportion of patients seen at the RASP continued their treatment with PHP? Measured by the number of patients fully discharged back into the care of their primary healthcare provider with treatment recommendations (chart review data).
  2. What proportion of patients seen at the RASP were referred to a community mental health program? Measured by the number of patients referred to a community mental health program by RASP psychiatrists (chart review data).
  3. What proportion of patients seen at RASP needed additional psychiatric evaluation at the RASP? Measured by the number of re-referrals to RASP by a PHP.
  4. What are the sociodemographic characteristics of the assisted population at program entry? Measured by the frequency and percentages of the sociodemographic characteristics (e.g. age, sex of birth, gender, ethnicity, employment status, source of income, income range, relationship status, family support, housing status, education, provincial zone) (chart review data).
  5. What is the status of patients’ recovery and health quality index, and the prevalence of likely major depressive disorder (MDD), likely generalized anxiety disorder (GAD), low resilience, childhood trauma, substance use, and suicide risk at program entry? Measured by patient-completed validated scales scores (chart review data).
  6. What is the prevalence of patients' diagnoses made by RASP psychiatrists at program entry? Measured by the frequency and percentages of each psychiatry diagnosis after psychiatry assessment (chart review data).
  7. What are the risk factors for ED presentation for mental health concerns and inpatient psychiatric treatments? Measured by Odds Ratios using logistic regression models with sociodemographic and clinical data from the assisted population one year before and after RASP assessment date.

### Hypothesis

**Fig. 2:**
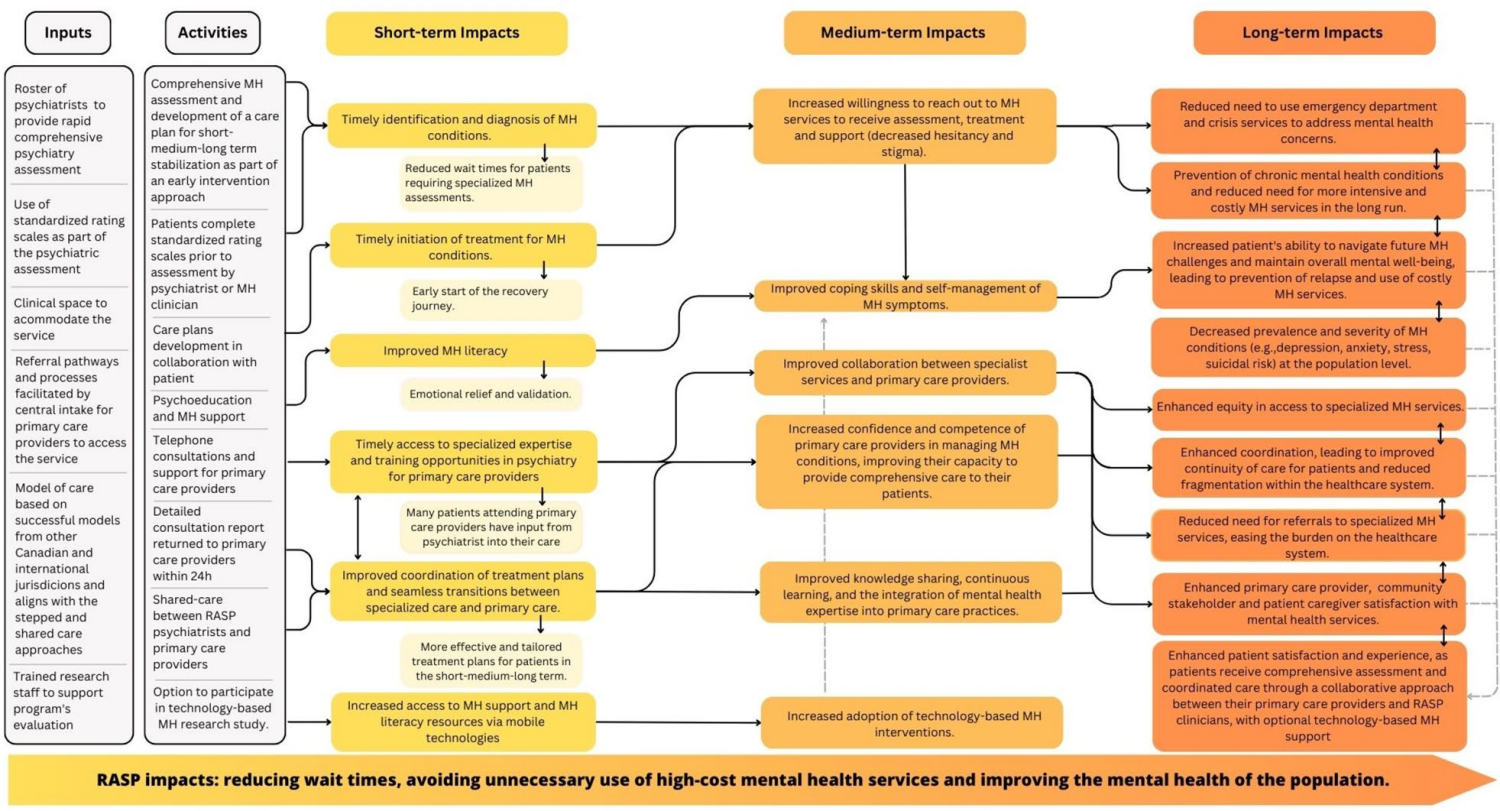
Logic Model for the RASP

Based on the project Logic Model (Fig. 2), we anticipate that the RASP initiative will yield positive impacts in the short, medium, and long terms, leading to substantial changes in the capacity of mental health support and primary health care providers. Specifically, we hypothesize that RASP will result in a minimum 10% reduction in high-cost health services utilization (e.g. ED visits, mobile crisis visits, and inpatient treatments) and corresponding 10% cost savings. Additionally, we anticipate a reduction in the wait times for patient consultations with psychiatrists within the publicly funded MHAP to less than 30 calendar days. This forecast is expected to be found in both within-subject and between-subject comparisons. In addition, we hypothesize that at least 90% of patients attending the RASP will express satisfaction with the services received. Finally, we hypothesize that primary care providers and healthcare partners will express high satisfaction with RASP. These hypotheses are derived from anecdotal evidence gathered from a similar program implemented at Fort McMurray between 2013 and 2016 by the Senior Investigator.

### Study design

**Fig. 3:**
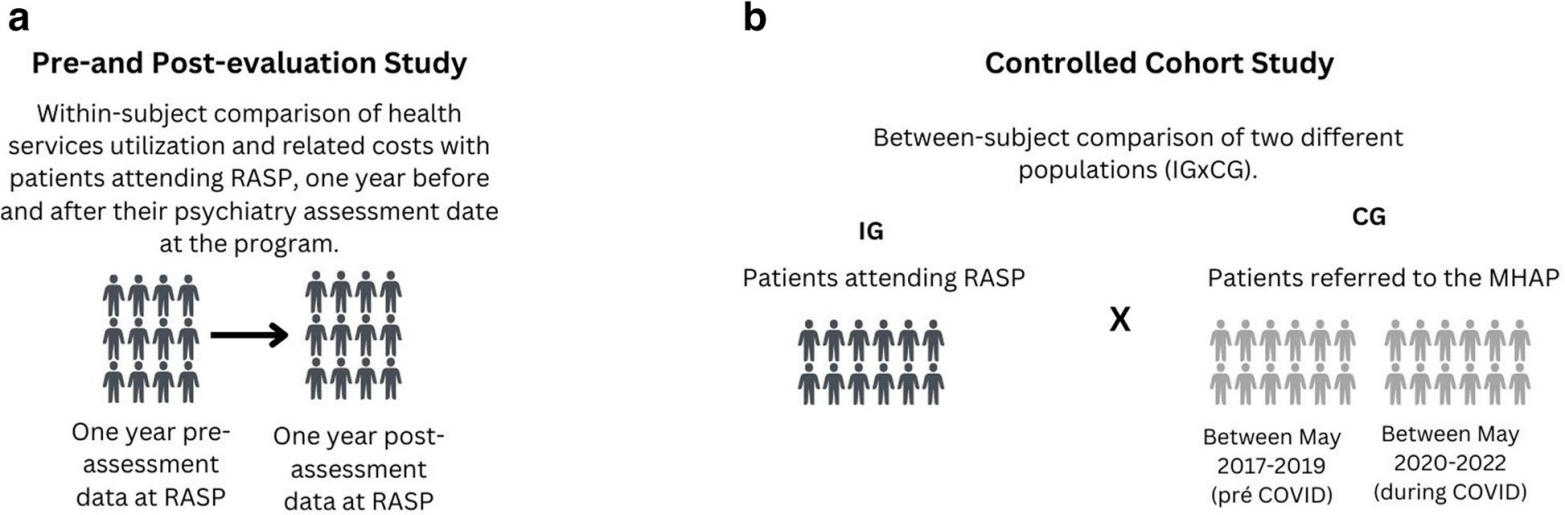
Study design diagram (**a** Pre-and post-evaluation study; **b** Controlled cohort study)

This is a hypothesis-driven program evaluation study that employs a mixed methods approach to evaluate the RASP. It consists of a within-subject comparison (pre- and post-evaluation study) and a controlled between-subject comparison (cohort study). The pre-and post-evaluation study will examine health services utilization data from patients attending RASP, one year before and one year after their psychiatry assessment at the program (Fig. 3a). The pre-and post-evaluation of health services utilization will also enable a comparison between RASP patients who choose to participate in the optional Text4Support RCT and those who choose not to participate in the optional Text4Support RCT.

The cohort study will compare two study populations: the intervention group (IG) consisting of patients attending RASP, and the control group (CG), comprising a cohort of patients who were referred by PHP to the MHAP central intake services prior to RASP implementation (Fig. 3b). This design allows researchers to examine whether possible changes in high-cost health services utilization are associated with the intervention (RASP).

A cross-sectional evaluation of mental health outcomes at program entry will be employed to evaluate the prevalence and correlates of the various psychological conditions of the assisted population.

Patient experience and satisfaction will be assessed using both quantitative and qualitative data. The quantitative data will be extracted from the program's satisfaction survey, while the qualitative data will be gathered from a purposive sub-sample of the overall study population. The selected sub-sample will be invited to participate in focus group discussions as part of a sub-study to gain deeper insights into their experiences and perspectives. Key informant interviews will be conducted to obtain qualitative insights from various healthcare partners, including directors, psychiatrists, primary healthcare providers, MHA service leaders, and community organizations. These interviews will focus on their perceptions regarding the impact of RASP on patients' access to community mental health care and the support it provides for primary care providers. Qualitative descriptive methodology and thematic analysis will be employed to generate and refine the information and identify patterns or themes within the qualitative data [35, 36]. This research protocol has been approved by the Research Ethics Board at Nova Scotia Health (REB file #1028254). The Gantt chart timeline is presented in the supplementary material (Table S1).

### Participants and data collection

All RASP patients receive a consent form as part of their intake process, allowing access to their health services utilization records. Only those who sign the consent form will be part of the study. While consent is obtained during the initial assessment for ongoing patient recruitment, data collection is scheduled to commence after the program's first year, in April 2024. Service utilization data will extracted from provincial information systems retrospectively and prospectively at 2-time points: one year pre- and one-year post-assessment date at RASP. In addition, sociodemographic and mental health assessment information, including results from patient-completed validated scales and psychiatry diagnosis, and patient satisfaction will be extracted from routine paper-based initial screening forms. Patient satisfaction quantitative data will be extracted from RASP's patient satisfaction survey, available in a paper-based format and completed immediately after the assessment or REDCap online survey link sent via text message to all patients attending the service just after the assessment appointment.

The control group for the cohort study will comprise patients referred to the MHAP Central Intake Services by a PHP before RASP implementation. The periods for inclusion in the control group will be May 2017 to May 2019 (pre-COVID) and May 2020 to May 2022 (during COVID). By examining the data from these two time points (pre-COVID and during COVID) and comparing them with each other and with data during the RASP implementation period, we will be able to evaluate the impact of the pandemic on health services utilization as well as the impact of RASP implementation in comparison with both the pre-pandemic and during the pandemic periods. Ethics approval for exemption from requiring informed consent for all control group patients has been granted by the Research Ethics Board at Nova Scotia Health (REB file #1028254), allowing control group patients' sociodemographic and health services utilization information from provincial administrative databases to be accessed by the investigators.

For the qualitative component of the study, expected to take place after a year of RASP implementation, in April 2024, a sub-sample from the overall pool of the intervention population (RASP patients) will be invited by a research team member to participate in a focus group session. The inclusion criteria to participate in the qualitative sub-study are to provide consent and be available to attend a 1-hour focus group session, which will be audio-recorded. The focus group script will be developed by investigators, reviewed by healthcare partners, piloted with patients and adjusted as needed, before being used in the focus groups. A trained research team member will conduct the encounters, which may happen in person or via Zoom. The sample size for the qualitative component of the study cannot be predetermined because data saturation needs to be reached. Data analysis ends when no new themes and subthemes emerge across all analysts. However, based on what the literature suggests [37], we anticipate that 25-30 participants may be sufficient for this mixed-methods study. Participants will receive compensation for their time spent participating in the qualitative sub-study activities.

Healthcare partners involved with RASP will be also actively engaged in the qualitative evaluation of the program. Trained members of the research team will conduct individual key informant interviews, either virtually or in person, with program directors, psychiatrists, primary healthcare providers, MHA service leaders, and representatives from community organizations. These interviews aim to gather in-depth information about healthcare partners' experiences and opinions regarding the program, allowing for a comprehensive understanding of their perspectives.

Finally, a sub-population of RASP patients who opted to participate in the Text4Support RCT will also have their one-year pre- and post-RASP attendance health services utilization data collected and compared to patients who chose not to participate in the optional Text4Support RCT. RASP was launched in April 2023, with data collection ongoing. Table S2 demonstrates in detail the variables, sources and data collection time points.

### Outcome measures

Primary outcome measures will include the mean differences in the frequency/duration/related costs of high-cost mental health services utilization (i.e., volume and proportion of mobile crisis visits, ED visits, hospital admissions and readmissions and associated LOS for each admission) between one year pre- and post-assessment date for same patients at RASP (within-subject comparison) and between the IG (RASP) and CG (historical data) (between-subjects comparison). Other primary outcome measures will be the mean difference in the number and proportion of total referrals booked directly for a psychiatrist and the number of calendar days for access to psychiatrists, also compared to the control group.

Secondary outcome measures include the mean difference in the frequency/duration/related costs of high-cost health services utilization one year post for patients who access the RASP and participate in the Text4Support RCT (to receive either daily supportive text messages or a single text message with a link to e-mental health resources) and patients accessing RASP but who do not participate in the Text4Support RCT. Other secondary outcome measures include a descriptive summary of the sociodemographic and clinical characteristics of the assisted population, the mean scores on the Patient Health Questionnaire 9 (PHQ-9) [38], Generalized Anxiety Disorder 7 (GAD-7) [39], World Health Organization 5 (WHO 5 Well-Being Index) [40], Brief Resilience Scale (BRS) [41], Recovery Assessment Scale (RAS) [42], Adverse Childhood Experience (ACE questionnaire) [43], Brief Substance use Craving Scale (BSCS) [44], and the Columbia-suicide severity rating scale (C-SSRS) [45], as well as the prevalence of likely MDD, GAD and low resilience. Patient overall impressions (i.e. barriers to accessing RASP, satisfaction level, likely recommend the service to others, etc.) and impressions on the experience (i.e. feelings of welcomeness, respect, enough time to talk, the problem addressed, and understanding of treatment) are part of the patient satisfaction quantitative evaluation. An in-depth evaluation of healthcare partner impressions and patient satisfaction will be obtained through qualitative key informant interviews until saturation is reached and no new themes and subthemes emerge.

### Sample size considerations

With two staff psychiatrists and a clinical coordinator, we expect an average of six patients to receive comprehensive psychiatric assessments at the RASP each working day. Thus, we anticipate that the total number of patients accessing the service over the 24-month data collection period would be at least 2500. We also expect the sample size for the control population (patients whom a primary care provider referred to the MHAP Central Intake Services between May 2020 and May 2022) to be at least equal to the numbers seen at the RASP over a similar two-year time frame. Therefore, the data set to be generated from an overall sample of 5000 patients in this study would be large. With the assumption that the implementation of RASP will lead to at least a 10% reduction in high-cost health services utilization one-year post initial referral from a primary care provider for patients attending the service compared to patients who were offered a future appointment to see a mental health clinician as an entry point to the community mental health program, a power of 90% (β = 0.1) and a two-sided significance level of α = 0.05, and assuming an aggregated mean high cost health services utilization of 10 (SD =1) for the control population, we estimated that a sample size of 525 per group would be sufficient. Given that we expect at least 2500 patients per group in our study, it is highly probable that our study has more than sufficient power to detect the projected differences in high-intensity/cost health services utilization between the two groups.

### Data analysis

#### Quantitative analysis

Quantitative data will be analyzed using descriptive and inferential statistics by using SPSS version 26 for Windows [46]. First, we will summarize the study participants' sociodemographic and clinical descriptive characteristics. Primary outcomes comparing the pre-and post-enrollment health services utilization for patients accessing RASP will be analyzed using descriptive and inferential statistical analysis, including Chi-Square and independent sample t-tests. For the primary outcome involving control populations, pre- and post-enrollment health services utilization variables will initially be compared using an analysis of covariance (ANCOVA) with the intervention condition (attended RASP) as the independent variable, the relevant health services utilization data one year pre attendance of appointment with RASP psychiatrist or community mental clinician as the covariate, and the relevant high-cost health services utilization data one year post attendance of appointment with a RASP psychiatrist or attendance with mental health clinicians in the community mental health program as the dependent variable. In each case, checks would be conducted to ensure no violation of regression slopes and reliable measurement of the covariate. We will perform sensitivity analyses of covariance to explore the impact of the imputation of data loss at each time point on health services utilization. In addition, we will perform propensity score matching and regression analyses adjusting for the periods pre-, during and post COVID-19 pandemic in order to strengthen the comparability of the groups and enhance the validity of the study's findings.

We will also use an ANCOVA to compare high-cost health services utilization data for one-year pre- and post-RASP attendance for the subset of patients who attend RASP and either participate or do not participate in the Text4Support RCT. For this analysis, the intervention condition (participants in the Text4Support RCT) will be the independent variable, the relevant health services utilization data one-year pre-attendance of appointment with RASP psychiatrist will be the covariate, and the relevant high-cost health services utilization data one-year post attendance of appointment with a RASP psychiatrist will be the dependent variable.

Using data from the first preliminary data analysis at the end of Year 2 and refining this at each subsequent analysis, we will perform regression to predict characteristics and risk factors for ED presentations for mental health concerns and inpatient psychiatric treatments [47]. For the categorical primary outcome measures, we will use the Chi-square test and multivariate logistic regression analysis to explore predictor variables for high-cost health services utilization variables of interest.

#### Health economic evaluation

We will conduct an economic evaluation through an effectiveness-implementation science lens. Specifically, we will conduct a cost-consequence analysis (CCA) from a Canadian single-payer perspective. The CCA will involve a disaggregated comparison of the costs and benefits (consequences) associated with RASP. The disaggregated analysis reports the intervention effects for the relevant primary and secondary outcomes in their natural units to aid value for money assessments. The study design, population, setting, and location are described elsewhere in this proposal.

We will compare outcomes (ED visits, crisis calls and inpatient days) and costs in the intervention group to the control group. Costing includes costs associated with the pre-implementation, implementation, and ongoing costs. These costs will include space and utilities, new hires specific to RASP, supplies, materials, and equipment, travel costs, volunteer time, and other in-kind donations. We will exclude all research-related costs. These cost data will come from the project documents. In addition, we will collect cost data on health resource use from the case costing centre in the Nova Scotia Health, supplemented with data from the literature if necessary.

Costing will also include costs saved from avoided ED visits, crisis response services and inpatient treatments. Cost analyses will use Nova Scotia Health 2023 costs to ensure relevance to decision-makers. We will estimate the intervention effect for the outcomes using appropriate treatment-effect econometric models with bootstrapping to quantify the uncertainty around the estimates. To enable cost comparisons, the cost of the current models of community-based mental health services, which involve waitlisting of all non-urgent patients (regardless of referral source and reason for referral) to see a mental health clinician as a gateway to receipt of community mental health services, including psychiatry, will be compared with the costs of alternative treatments offered expeditiously through RASP and primary care and community providers. We will compute the net cost savings associated with RASP. Our economic analysis will follow guidelines for economic evaluations, including using probabilistic sensitivity analysis to quantify the uncertainty around the implementation costs [48].

#### Qualitative analysis

Aligned with qualitative descriptive methods, the qualitative data analysis will be guided by the six-phase thematic analysis framework [36]. We will transcribe verbatim all audio records from the semi-structured individual interviews and focus groups and enter them in NVIVO 12 [49] software for data organization and preparation for analysis.

The first step of qualitative analysis is to become familiar with the data, reading and re-reading the transcripts. At this stage, we will also perform data validation for quality assurance purposes. A data validator, who is a separate individual from the transcriber, will compare the verbatim transcriptions with the recorded interviews for verbal and non-verbal errors (commissions and omissions). After that, the subsequent phases of thematic analysis (coding, searching for themes, themes review, themes definitions, and writing) will be completed [36]. As previously mentioned, the sample size for the qualitative sub-study will depend on data saturation [37].

## Conclusion

This study will demonstrate the results of the NSH/MHAP efforts and commitment to providing early intervention services within the stepped-care model, particularly early access to psychiatrists for diagnostic clarification, medication initiation or adjustments, and connections to community-based support for individuals with mental illnesses. Findings of this program evaluation will provide evidence of new community-based approaches to mental health service delivery and contribute to knowledge on how to implement mental health programs across varying contexts.

### Supplementary Information

**Additional file 1: Table S1.** Gantt chart timeline for Study 1.

**Additional file 2: Table S2.** Outcomes, study variables, data sources and data collection time points.

## Data Availability

Not applicable.

## Abbreviations

RASP: Rapid Access and Stabilization Program
MHAP: Mental Health and Addictions Program
ED: Emergency Department
LOS: Length of Stay
PHP: Primary Healthcare Providers
NSH: Nova Scotia Health
RCT: Randomized Controlled Trial
CMHP: Community Mental Health Programs
MHA: Mental Health and Addictions
MDD: Major Depressive Disorder
GAD: Generalized Anxiety Disorder
IG: Intervention Group
CG: Control Group
PHQ-9: Patient Health Questionnaire 9
GAD-7: Generalized Anxiety Disorder
WHO-5: World Health Organization Well-Being Index
BRS: Brief Resilience Scale
RAS: Recovery Assessment Scale
ACE: Adverse Childhood Experience questionnaire
BSCS: Brief Substance use Craving Scale
C-SSRS: Columbia-Suicide Severity Rating Scale
CCA: Cost-consequence Analysis
