# Supplementary material for: Reducing wait times and avoiding unnecessary use of high-cost mental health services through a Rapid Access and Stabilization Program: protocol for a program evaluation study
Source: BMC Health Serv Res. 2024 Feb 27;24:247. doi: 10.1186/s12913-024-10697-7 (PMC10898149; doi:10.1186/s12913-024-10697-7)
Supplement: Supplementary file 1 — Additional file 1: Table S1. Gantt chart timeline for Study 1. [file 12913_2024_10697_MOESM1_ESM.docx]

***Table S1: Gantt chart timeline for Study 1***

| **Milestones** | | **Year 1** | | | | **Year 2** | | | | **Year 3** | | | |
| --- | --- | --- | --- | --- | --- | --- | --- | --- | --- | --- | --- | --- | --- |
|  |  | **Q1** | **Q2** | **Q3** | **Q4** | **Q1** | **Q2** | **Q3** | **Q4** | **Q1** | **Q2** | **Q3** | **Q4** |
| **Milestone 1: Logistic preparations** | | | | | | | | | | | | | |
| 1.1 | Development of electronic data abstraction form and data collection workflow. | X |  |  |  |  |  |  |  |  |  |  |  |
| 1.2 | Training of research team members in data abstraction | X |  |  |  |  |  |  |  |  |  |  |  |
| **Milestone 2: Pre-post Data collection** | | | | | | | | | | | | | |
| 2.1 | Sociodemographic, clinical, quality of care, services utilization and satisfaction secondary data collection. |  | X | X | X | X | X | X | X | X | X |  |  |
| **Milestone 3: Qualitative data collection** | | | | | | | | | | | | | |
| 3.1 | Key informant/examiner partner interviews and focus groups to collect patient experience and qualitative satisfaction data |  |  | X |  |  | X |  |  |  |  |  |  |
| **Milestone 3: Data compilation, analysis and preparation of reports, publications and presentations for multi-scale dissemination** | | | | | | | | | | | | | |
| 4.1 | Data compilation. |  |  |  | X | X | X | X | X | X | X | X | X |
| 4.2 | Data analysis. |  |  |  | X | X | X | X | X | X | X | X | X |
| 4.3 | Preparation of reports, publications and presentations. |  |  |  |  |  |  | X | X | X | X | X | X |
