# Supplementary material for: Reducing wait times and avoiding unnecessary use of high-cost mental health services through a Rapid Access and Stabilization Program: protocol for a program evaluation study
Source: BMC Health Serv Res. 2024 Feb 27;24:247. doi: 10.1186/s12913-024-10697-7 (PMC10898149; doi:10.1186/s12913-024-10697-7)
Supplement: Supplementary file 2 — Additional file 2: Table S2. Outcomes, study variables, data sources and data collection time points. [file 12913_2024_10697_MOESM2_ESM.docx]

**Table S2:** Outcomes, study variables, data sources and data collection time points

| **Outcomes** | **Study variables** | **Data source** | **Data collection time points** | | | | | |
| --- | --- | --- | --- | --- | --- | --- | --- | --- |
|  |  |  | **Pre-and post-evaluation study** | | **Controlled cohort study** | | **Cross-sectional evaluation** | **Qualitative sub-study** |
|  |  |  | 1 year pre-assessent date at RASP | 1year post-assessment  date at RASP | May 2017 to May 2019 | May 2020 to May 2022 | At assessment date at RASP | At focus group session or key informant interview |
| ***Variables of Impact on high-cost health services utilization*** | | | | | | | | |
| Volume of patients referred directly to a psychiatrist | Number of patients referred by a PCP with request/indication to book a consultation directly with a psychiatrist. | Administrative data from provincial databases | X | X | X | X |  |  |
| Proportion of overall patients triaged by Central Intake referred directly to a psychiatrist | Number of referrals received by central intake that were booked directly for psychiatry consultation divided by total volume of referrals received. | Administrative data from provincial databases | X | X | X | X |  |  |
| Wait times to see a psychiatrist | Number in calendar days from when a PCP referral was received by Central Intake that patients had to wait for access a psychiatrist. | Administrative data from provincial databases | X | X | X | X |  |  |
| High-cost health services utilization | Cost of MHA-specific ED visits, mobile crisis visits, and MHA-specific hospital admissions, | Administrative data from provincial databases | X | X | X | X |  |  |
| Net cost savings | Costs associated with the program and costs saved from avoided high-cost health services utilization (ED visits, mobile crisis visits, and inpatient treatments). | Administrative data from provincial databases | X | X | X | X |  |  |
| ***Variables of healthcare partners perceptions*** | | | | | | | | |
| Overall impressions and perceptions with the program | Qualitative data | Key information interviews with program directors, psychiatrists, primary healthcare providers, MHA service leaders, and representatives from community organizations. |  |  |  |  |  | X |
| ***Variables of patient experiences and satisfaction*** | | | | | | | | |
| Overall impressions and level of satisfaction with the service | Quantitative data | RASP's satisfaction survey |  |  |  |  |  | X |
| Deep understanding of patient experience and satisfaction with the service | Qualitative data | Focus group sessions with a sub-group of the assisted population and RASP’s satisfaction survey. |  |  |  |  |  | X |
| ***Variables of population characteristics*** | | | | | | | | |
| Proportion of patients in need of a re-referral after RASP assessment | Number of re-referrals to RASP by a PHP. | Administrative data from provincial databases and medical chart review |  | X |  |  |  |  |
| Proportion of patients who continued their treatment with a PHP | Number of patients fully discharged back into the care of their primary healthcare provider with treatment recommendations | Administrative data from provincial databases and medical chart review |  | X |  |  |  |  |
| Proportion of patients referred to a community mental health program after RASP assessment | Number of patients referred to a community mental health program by RASP psychiatrists | Administrative data from provincial databases and |  | X |  |  |  |  |
| Sociodemographic characteristics | Age, sex of birth, gender, ethnicity, employment status, source of income, income range, relationship status, family support, housing status, education, provincial zone. | Medical chart review |  |  |  |  | X |  |
| Mental health status | Patient-completed validated scales scores (Patient Health Questionnaire 9 (PHQ-9) [37], Generalized Anxiety Disorder 7 (GAD-7) [38], World Health Organization 5 (WHO 5 Well-Being Index) [39], The Brief Resilience Scale (BRS) [40], Recovery Assessment Scale (RAS) [41], Adverse Childhood Experience (ACE questionnaire) [42], and Brief Substance use Craving Scale (BSCS) [43]). | Medical chart review |  |  |  |  | X |  |
| Mental health status (psychiatrist determined) | Clinician-completed Columbia-Suicide Severity Rating Scale (C-SSRS) [44], and psychiatry diagnosis after assessment. | Medical chart review |  |  |  |  | X |  |
